# Efficacy and safety of acupuncture monotherapy or combined with pelvic floor muscle training for female stress urinary incontinence: a systematic review and meta-analysis

**DOI:** 10.3389/fmed.2024.1499905

**Published:** 2025-01-13

**Authors:** Tong Jiang, Zhi-Yu Dong, Ying Shi, Yang-Qing Zhou, Hong-Bin Zhang, Yi Gong

**Affiliations:** ^1^Beilun District People’s Hospital, Beilun Branch of the First Affiliated Hospital of Zhejiang University, Ningbo, China; ^2^Taizhou Traditional Chinese Medicine Hospital, Taizhou, China

**Keywords:** stress urinary incontinence, electroacupuncture, pelvic floor muscle training, urine leakage, international consultation on incontinence questionnaire - short form

## Abstract

**Background:**

Stress urinary incontinence (SUI) is involuntary urine leakage during effort. Pelvic floor muscle training (PFMT) is a common physical therapy for SUI, but has low adherence and its long—term effectiveness is uncertain. Drug therapy has side-effect problems and surgery has risks. Acupuncture’s role in improving SUI is also unclear based on previous studies.

**Objectives:**

This systematic review and meta-analysis evaluated the efficacy and safety of acupuncture individually or combined with PFMT in improving symptoms and women’s health-related quality of life of SUI.

**Design:**

A systematic review and meta-analysis were conducted.

**Patients:**

Women with stress urinary incontinence were treated with electroacupuncture (EA) individually or acupuncture combined with PFMT.

**Results:**

In the analysis comparing urinary leakage 1 h and the ICIQ-SF score, electroacupuncture (EA) intervention was significantly associated with improvements in both urine leakage (MD = 4.69, 95% CI 2.83 to 6.55, *I^2^* = 62%) and ICIQ-SF (MD = 2.61, 95% CI 1.64 to 3.58, *I^2^* = 65%). The results were robust for the sensitivity analyses. EA was not associated with an increased incidence of adverse events compared to placebo EA (RR = 1.08, 95% CI 0.50 to 2.35, *I^2^* = 0%). When comparing the group receiving pelvic floor muscle training (PFMT) combined with acupuncture to the PFMT only group, the combination therapy was found to improve urine leakage 1 h (MD = 1.91, 95% CI 0.96 to 2.86, *I^2^* = 80%) and improve ICIQ-SF (MD = 2.63, 95% CI 1.60 to 3.65, *I^2^* = 75%) in patients with SUI, despite significant heterogeneity observed. Subgroup analyses based on urine leakage 1 h revealed that subjects with mild stress urinary incontinence showed improvements (MD = 1.46, 95% CI 0.82 to 2.10, *I^2^* = 58%), as did those with moderate stress urinary incontinence (MD = 4.9, 95% CI 1.72 to 8.08, *I^2^* = 77%). There were significant differences between these subgroups (*I^2^* = 77%, *p* = 0.04). In the subgroup analysis of intervention types, manual acupuncture showed no significant effect when combined (MD = 1.11, 95% CI -0.61 to 2.83, *I^2^* = 86%).

**Conclusion:**

The findings from this meta-analysis indicate that EA is more effective at improving the clinical symptoms and quality of life in patients with SUI compared to placebo EA, and it does not increase the risk of adverse events. Moreover, the therapeutic effect of SUI treatment with EA combined with pelvic floor muscle training (PFMT) elicits a more positive response than PFMT alone. This suggests that EA, either as a standalone therapy or as an adjunct to PFMT, can offer beneficial outcomes for individuals with stress urinary incontinence, expanding the range of clinical treatment options available.

**Systematic Review Registration:**

The meta-analysis was registered in PROSPERO (CRD42022381409).

## Graphical abstract

Created in BioRender. YI, G. (2024) https://BioRender.com/f58r840.

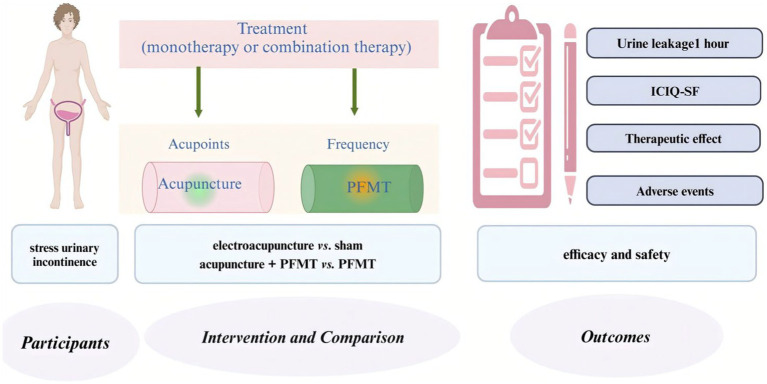

## 1 Introduction

The IUGA and ICS classify urinary incontinence (UI) as involuntary urine loss, with main types being stress (SUI), urgency (UUI), and mixed (MUI) incontinence. UI’s causes often relate to age, gender, BMI, and vaginal delivery history. Global UI rates differ by country and diagnostic practices. In China, adult female UI prevalence ranges widely, from 8.7 to 69.8% (1, 2). Treatment for SUI can encompass a variety of approaches, with the choice of therapy typically depending on the severity of symptoms, patient preferences, and willingness to undergo surgical treatment. The main treatment options include lifestyle modifications, Pelvic Floor Muscle Training (PFMT), physical therapy, pharmacological management, and surgical interventions.

Multiple guidelines, including those from the American College of Obstetricians and Gynecologists (ACOG), the International Consultation on Incontinence (ICI), and the National Institute for Health and Care Excellence (NICE) in the UK, mention Kegel exercises (Pelvic Floor Muscle Training) as an effective part of non-surgical treatment to help improve urinary incontinence (3–5). PFMT can strengthen the pelvic structure, yet many individuals with SUI struggle to execute PFMT properly, and the main challenge is maintaining long-term adherence to the treatment (6).

Drug therapy causes problems such as side effects and surgery exists associated with risks (7, 8). Expanding the currently available treatment options, and developing individualized treatment plans for patients according to a woman’s lifestyle, expectations, and goals for treatment, as well as her tolerance for potential adverse events is becoming increasingly important (9). Acupuncture may improve urinary incontinence through its effects on neural regulation (10), enhancing the pelvic floor muscles (6). Both electroacupuncture(EA) and manual acupuncture can effectively improve stress urinary incontinence, and older women and women after childbirth can also benefit from acupuncture treatment (11–14). However, the Cochrane review published in 2013 and 2022 believed that the evidence for the effect of acupuncture on stress urinary incontinence or improvement of overactive bladder symptoms was not clear, and the conclusions should be used with caution due to the generally low quality of the included studies (15, 16).

Electroacupuncture has been proven effective in improving stress urinary incontinence, but research has been limited. This systematic review and meta-analysis evaluate the effectiveness and safety of electroacupuncture in treating stress urinary incontinence and further assesses the efficacy of combining acupuncture with conventional treatment (PFMT) as a supplemental therapy.

## 2 Materials and methods

The meta-analysis was registered in PROSPERO (CRD42022381409) and prepared according to preferred reporting items of systematic reviews and meta-analysis (PRISMA) (17).

### 2.1 Data sources and literature search

A comprehensive literature search was conducted from inception to January 2023, including databases such as PubMed, Embase, Cochrane, and CNKI. In the search strategy, a systematic approach was utilized with the following Medical Subject Headings (MeSH) and free-text keywords. The studies were not restricted to those published in English. Additionally, the references of related documents were searched to ensure their identification as comprehensively as possible.

### 2.2 Inclusion and exclusion criteria

Inclusion criteria: (1) Participants: Women with stress urinary incontinence. (2) Interventions: electroacupuncture, acupuncture combined with PFMT. Types of interventions acupuncture therapies include penetrating the skin (acupuncture, electroacupuncture, and warm needling), control acupuncture (non-penetrating the skin), and PFMT individually; (3) Study design: Designed as a randomized controlled trial (RCT).

Exclusion criteria: (1) We excluded mixed incontinence and other types of incontinence; (2) Studies with additional interventions other than acupuncture or PFMT were excluded.

### 2.3 Study selection

The literature screening was done independently by two authors (TJ, ZYD), Firstly screening the title and abstract, eliminating the literature that does not meet the requirements, and further reading the full text to screen literature. After the data extraction a meta-analysis was performed. Any disagreement was resolved by discussion until consensus was reached or by a third author (YG).

### 2.4 Data extraction and outcome measures

Two independent reviewers (TJ, ZYD) extracted the data from the included studies. The extracted data included the first author’s name, year of publication, sample, participant characteristics (screening tools, age, severity), intervention characteristics, control characteristics, therapy duration, study outcomes. Any discrepancies were addressed through discussion, and consensus was achieved among the reviewers.

Primary outcome: Urine leakage 1 h (by urinal pad test), international consultation on incontinence questionnaire short form (ICIQ-SF), adverse events.

Secondary outcomes: 72-h incontinence episodes. Self-report (self-evaluation of therapeutic effects, severity of stress urinary incontinence).

SUI severity was defined based on baseline levels of urine leakage (mild, 1.1–9.9 g; moderate, 10–49.9 g; severe, ≥50 g) (18). To include a greater number of studies, the mean baseline levels were taken into consideration.

### 2.5 Risk of bias assessment

The Risk of bias tool (19) was used to evaluate the quality of included studies. The items were as follows: (1) Random sequence generation (selection bias); (2) Allocation concealment (selection bias); (3) Blinding of participants and personnel (performance bias); (4) Blinding of outcome assessment (detection bias); (5) Incomplete outcome data (attrition bias); (6) Selective reporting (reporting bias); (7) Other bias. Quality assessments were performed independently by two authors. Any disagreements are resolved by a third investigator.

### 2.6 Data analysis

Review Manager 5.3 and STATA version 16.1 (Stata Corp, College Station, Texas, USA), and SAS (Statistics Analysis System9.4, USA) were used to perform meta-analysis.

For continuous variables, we calculated the effect sizes as mean difference (MD) and 95% confidence interval (CI) between the acupuncture intervention and control arms. Categorical variables were pooled as risk ratio (RR) and 95% CI. For ordered categorical variables, effect size (logor) and standard error (selogor) were calculated by the cumulative ratio model and the generic inverse variance method was used to calculate OR and 95% CI (20, 21).

The random-effect model was employed by using Inverse Variance Method when the study of heterogeneity (*I^2^*) was larger than 50%; otherwise, a fix-effect model was employed by using the inverse variance method when the *I^2^* was less than 50%.

The data reported by interquartile range for continuous outcomes was transformed into mean and standard deviation (22, 23), when it was necessary. The data reported as means and 95% confidence intervals were transformed by the following formula.


$$SD=\frac{95\%CI-\mathrm{mean}}{Z_{\alpha /2}/\sqrt{n}}$$

The data reported only in the form of means and standard deviations at baseline and after treatment are transformed by the following formula.

$$SD1\left(C\right)=\sqrt{SD_{1}{\left(B\right)}^{2}+SD_{1}{\left(F\right)}^{2}-\left(2\times R_{1}\times SD_{1}\left(B\right)\times SD_{1}\left(F\right)\right)¨\text{.}}$$

Subgroup analyses include population (diagnostic criteria, severity, age) and intervention (intervention types, acupoints belong to, duration).

## 3 Results

### 3.1 Literature search

**Figure 1:**
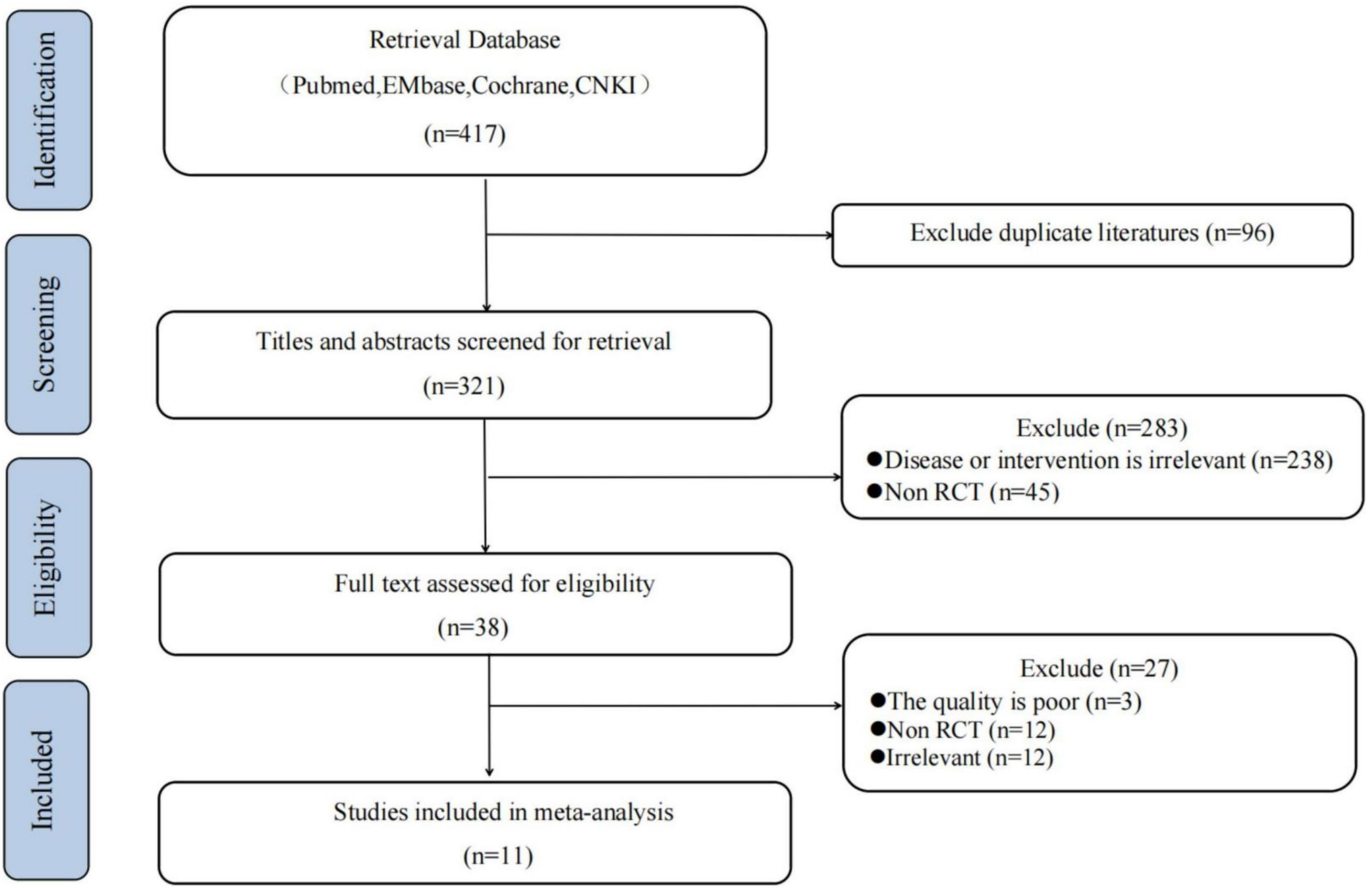
Flow chart of study.

There were 417 studies retrieved from the database searches, and 96 duplicates were eliminated. Two reviewers conducted screening of titles and abstracts and full-text assessment independently. Ultimately, 11 studies were included in this review and meta-analysis. (Figure 1 and Supplementary Figure S2).

### 3.2 Study characteristics

Eleven studies were included in the analysis, published between 2017 and 2022, with sample sizes ranging from 42 to 504. All studies originated from China, with five studies comparing electroacupuncture (EA) with sham electroacupuncture (placebo EA), and six studies comparing acupuncture or manual acupuncture combined with PFMT with PFMT alone. The specific details are shown in Table 1.

**Table 1:** Characteristics of the studies included.

	Author (year)	Sample size	Population	Intervention			Outcome
		Intervention/Control	Screening tools/ Age/Severity	Acupuncture group	Control group	Duartion	
1	Zhishun (11) (2017)	I = 252 C = 252	1. International Consultation on Incontinence(5th) 2. 40–75 years 3. Mild, moderate and severe	Electroacupuncture: 1. Acupoints BL33, BL35 2. Parameters:50 Hz;1-5 mA	Sham electroacupuncture: 1. Acupoints:20 mm lateral to BL33 and BL35. 2. Parameters: no electric output without skin penetration	42 days (three times a week)	1. 1-h pad test ^#^; 2. 72-h incontinence episodes #; 3. ICIQ-SF ^#^ 4. Volume of liquid intake * 5. Use of urine pads* 6. Self-evaluation of therapeutic effects * 7. Adverse events *
2	Kangmin (24) (2022)	I = 150 C = 154	1. International Consultation on Incontinence (5th) 2. 40–75 years 3. Mild, moderate and severe	Electroacupuncture combine with PFMT: 1. Acupoints BL33, BL35 2. Parameters 50 Hz;1-5 mA	Sham electroacupuncture combine with PFMT: 1. Acupoints, BL33 and BL35. 2. Parameters, without skin penetration	1. Electroacupuncture: 56 days (three times a week) 2. PFMT:56 days (three times a day)	1. Therapeutic effect * 2. 1-h pad test ^#^ 3. ICIQ-SF ^#^ 4. Adverse events *
3	Huanfang (25) (2016)	I = 40 C = 40	1. International Consultation on Incontinence (4th) 2. 40–75 years 3. Mild, moderate and severe	Electroacupuncture: 1. Acupoints, BL33, BL35 2. Parameters, 50 Hz;1-5 mA	Sham electroacupuncture: 1. Acupoints, 20 mm lateral to BL33 and BL35. 2. Parameters, no electric output without skin penetration	42 days (three times a week)	1. 1-h pad test ^#^ 2. ICIQ-SF ^#^ 3. 72-h incontinence episodes ^#^; 4. Self-evaluation of therapeutic effects * 5. Adverse events *
4	Qianhuan (27) (2021)	I = 21 C = 21	1. International Consultation on Incontinence (4th) 2. 35–65 years 3. Mild and moderate	Electroacupuncture: 1. Acupoints: BL33, BL35 2. Parameters:50 Hz; 2–6 mA	Sham electroacupuncture: 1. Acupoints:20 mm lateral to BL33 and BL35. 2. Parameters:no electric output without skin penetration	42 days (three times a week)	1. 1-h pad test * 2. 72-h incontinence episodes *
5	Chunxiao (26) (2017)	I = 38 C = 38	1. International Consultation on Incontinence (4th) 2. 40–75 years 3. Mild and moderate	Electroacupuncture: 1. Acupoints: BL33, BL35 2. Parameters:50 Hz; 1–5 mA 3. PFMT	Sham electroacupuncture: 1. Acupoints:20 mm lateral to BL33 and BL35. 2. Parameters:no electric output without skin penetration 3. PFMT	1. 56 days (three times a week) 2. PFMT:56 days (three times a day)	1. 1-h pad test * 2. ICIQ-SF * 3. Therapeutic effect *
6	Ding (29) (2022)	I = 32 C = 32	1. Guidelines for the diagnosis and treatment of stress urinary incontinence in women (2017), CHINA 2.42–75 years 3. Mild	Electroacupuncture combine with PFMT: 1. Acupoints: BL20, BL23, BL31, BL32, BL33, BL34 2. Parameters:30 Hz; 1-5 mA	PFMT	1. Electroacupuncture: 60 days (five times a week) 2. PFMT:60 days (twice a day)	1. Mean urine leakage in 24 h * 2. Therapeutic effect * 3. 1-h pad test * 4. ICIQ-SF *; 5. Pelvic floor muscle strength score *
7	Huijie (30) (2021)	I = 20 C1 = 20 C2 = 20	1. Guidelines for Diagnosis and Treatment of Stress Incontinence in Women of Peking University (Draft), CHINA 2.20–70 years 3. Mild and moderate	Traditional acupuncture combine with PFMT: Acupoints: BL28, BL23, CV2, CV3, CV4, CV6, ST28, SP6	PFMT	1. Acupuncture: (28 days, six times a week); 2. PFMT:28 days (daily, six times a week)	1. Therapeutic effect* 2. 1-h pad test* 3. ICIQ-SF* 4. Pelvic floor muscle strength score*
8	Jun (31) (2020)	I = 30 C = 30	Guidelines for the diagnosis and treatment of stress urinary incontinence in women (2017), CHINA 25–65 years Moderate	Electroacupuncture combine with PFMT.: 1. Acupoints: BL20, BL21, BL22, BL23 2. Parameters:2HZ	PFMT	1. Electroacupuncture: 84 days (3 times a week) 2. PFMT:84 days (three times a day)	1. Therapeutic effect * 2. 1-h pad test * 3. IIQ-7*
9	Yanming (28) (2020)	I = 80 C = 80	1. International Consultationon Incontinence(4th) 2. 40–65 years 3. Mild	Warm acupuncture combine with PFMT: Acupoints: GV2, GV3, GV20, CV3, CV4, CV8	PFMT	Acupuncture: 56 days (three times a week,) PFMT:56 days (three times a day)	1. Therapeutic effect * 2. 1-h pad test * 3. ICIQ-SF * 4. Urodynamics *
10	Wanzhen (32) (2019)	I = 30 C = 30	1. Guidelines for the diagnosis and treatment of stress urinary incontinence in women (2017), CHINA 2. 40–70 years 3. Moderate	Electro-warming acupuncture 1. Acupoints: BL31, BL32, BL33 and BL34 2. Parameters: UK	PFMT	Electroacupuncture: 40 days (once every other day) PFMT 40 days (three times a day)	1. Therapeutic effect * 2. 1-h pad test * 3. ICIQ-SF* 4. post - void residual urine volume *
11	Wenguang (33) (2017)	I = 40 C = 42	1. International Continence Society 2. 30–75 years 3. Mild and moderate	Electroacupuncture combine with PFMT: 1. Acupoints: BL23, BL35, BL32, CV4, CV3, SP6, KI12 2. Parameters: 4–20 Hz	PFMT	Electroacupuncture: 28 days (once every other day) PFMT:28 days (three times a day)	1. Therapeutic effect * 2. 1-h pad test * 3. ICIQ-SF*

IIQ-7, incontinence impact questionnaire short form. ICIQ-SF: International Consultation on Incontinence Questionnaire Short Form VAS: Visual Analogue Scale UDI: Urogenital Distress Inventory. ^#^Change from baseline, *After treatment. Channels and collaterals, BL (Bladder Meridian), CV(Conception Vessel), GV(Governing Vessel), SP(Spleen Meridian), KI(Kidney Meridian).

### 3.3 Quality assessment of the included studies

**Figure 2:**
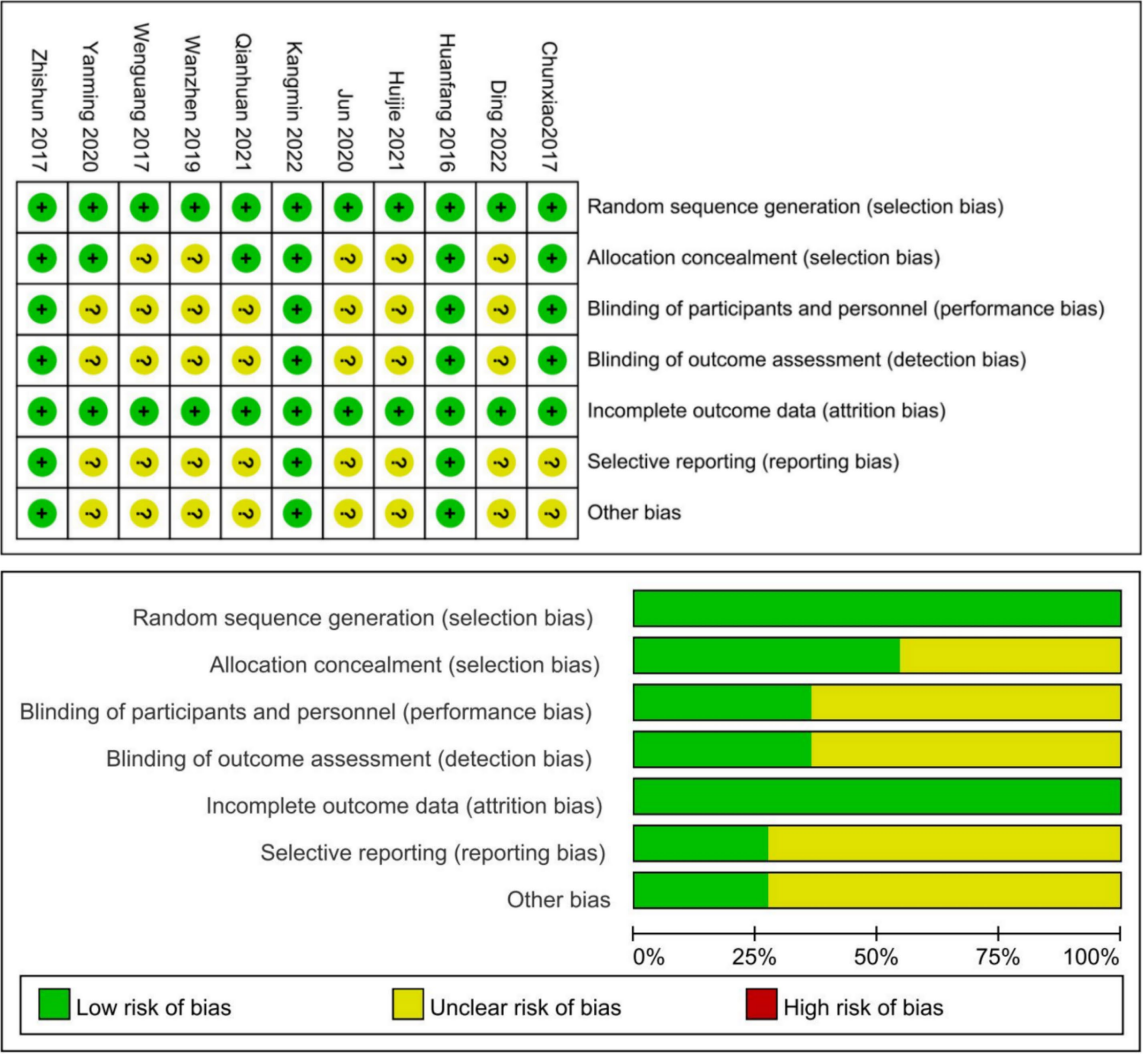
Risk of bias of the included studies.

11 studies reported a random sequence generation method, six studies used a central randomization system or block randomization (11, 24–28), and in five studies randomization was done with the use of a random digit table (29–33). Six studies reported concealment of the allocation (11, 24–28)^.^ Four studies describe blinded method, one study was single-blinded (26), three were double-blind (11, 24, 25)^.^ Four studies reported blinding of outcome assessors (11, 24–26). No studies were found to have incomplete outcome data. Only three studies were registered on ClinicalTrials.gov (11, 24, 25), and we assessed them without selective reporting and other biases, all other studies were of unknown risk (Figure 2).

### 3.4 Results

In the comparison of Urine leakage1 hour and ICIQ-SF, pooled five high-quality studies results showed that (11, 24–27), there was a significant association between EA intervention and improvement in Urine leakage compared to sham EA (MD = 4.69, 95% CI 2.83 to 6.55, *I^2^* = 62%). Compared with sham EA, EA can significantly improve ICIQ-SF in SUI patients (11, 24–26) (MD = 2.61, 95% CI 1.64 to 3.58, *I^2^* = 65%). The results were robust for the sensitivity analyses. A comparison of the three studies found no heterogeneity in significant differences in patients’ self-reports of therapeutic effects (OR = 10.46, 95% CI 7.81 to 14.01, *I^2^* = 0%).

In the comparison of adverse events, the data of 3 studies showed that EA did not increase the incidence of adverse events compared with placebo EA (RR = 1.08, 95% CI 0.50 to 2.35, *I^2^* = 0%).

In a comparison of PFMT combined with acupuncture group and PFMT individually group, a total of 6 low-quality studies showed (28–33) that compared with PFMT individually, the combined use of acupuncture can improve the urine leakage 1 h in SUI patients, but there is obvious heterogeneity (MD = 1.91, 95% CI0.96 to 2.86, *I^2^* = 80%). Five studies showed that the combined use of acupuncture could improve ICIQ-SF of SUI patients (28–30, 32, 33) (MD = 2.63, 95% CI1.60 to 3.65, *I^2^* = 75%). The results were robust for the sensitivity analyses (Supplementary Figure S1). The comparison of four studies found no heterogeneity of significant differences in patients’ self-reported therapeutic effects (OR = 3.79, 95% CI 2.39 to 6.00, *I^2^* = 0%) (28–31).

Subgroup analyses

The results of subgroup analyses by urine leakage 1 h are summarized as follows, in the three studies (28, 29, 33) that included subjects who tended to have mild stress urinary incontinence (MD = 1.46, 95% CI 0.82 to 2.10, *I^2^* = 58%). In the three studies that tended to have moderate stress urinary incontinence, study heterogeneity increased and effect size increased (MD = 4.9, 95% CI 1.72 to 8.08, *I^2^* = 77%) (31–33). There were also significant differences between subgroups (*I^2^* = 77%, *p* = 0.04). In the subgroup analysis of different intervention types, the two studies of manual acupuncture were combined (28, 30) and no significant effect was found (MD = 1.11, 95% CI –0.61 to 2.83, *I^2^* = 86%). In the subgroup analysis of the acupoints, the two studies involving the bladder meridian and others were combined (30, 33) and no significant effect was found (MD = 2.58, 95% CI –0.28 to 5.43, *I^2^* = 88%).

The results of subgroup analyses by ICIQ-SF are summarized as follows, in the subgroup analysis of different intervention types, the two studies of manual acupuncture were combined (28, 30) and no significant effect was found(MD = 1.47, 95% CI –1.39 to 4.33, *I^2^* = 93%), and 20–75 years old in the age subgroup (30, 33) no significant effect was found (MD = 2.10, 95% CI –0.15 to 4.34, *I^2^* = 85%). In the subgroup analysis of acupoints, the two studies of bladder meridian combined with others were combined (30, 33) and no significant effect was found (MD = 2.10, 95% CI –0.15 to 4.34, *I^2^* = 85%) (Tables 2–4).

**Table 2:** Meta-analysis for electroacupuncture.

Comparison of electroacupuncture group and sham electroacupuncture group								
Sample			Heterogeneity		Mode used	Meta-analysis		
Outcomes	Studies	Patients	*I^2^*%	*p*		Mean Difference Odd/Risk Ration	95%CI	*p*
1. Urine leakage1 hour*	5	990	62	0.03	R	4.69	(2.83, 6.55)	0.00
2. Reduction ≥50% urine leakage**	2	786	75	0.04	R	2.49	(1.77, 3.50)	0.00
3. ICIQ-SF*	4	938	65	0.04	R	2.61	(1.64, 3.58)	<0.00
4. 72-h incontinence episodes*	2	562	0	0.77	F	1.01	(0.31, 1.71)	0.00
5. Adverse events**	3	877	0	0.84	F	1.08	(0.50, 2.35)	0.85
6. Self-reported***								
Self-evaluation of therapeutic effects	3	877	0	0.96	F	10.46	(7.81, 14.01)	<0.00
Severity of stress urinary incontinence	2	786	80	0.02	R	2.19	(1.13, 4.26)	0.02

*Data are presented *p*-values for continuous variables comparison (Mean Difference). **Data are presented p-Values for Categorical variables comparison (Risk Ration). ***Data are presented *p*-values for ordered categorical variables comparison (Odds Ration).

**Table 3:** Meta-analysis for PFMT combine with acupuncture.

Comparison of PFMT combine with acupuncture group and PFMT individually group								
Sample			Heterogeneity		Mode used	Meta-analysis		
Outcomes	Studies	Patients	*I^2^*%	*p*		Mean Difference Odd/Risk Ration	95%CI	*p*
(A) Urine leakage1 hour*	6	466	80	<0.00	R	1.91	(0.96,2.86)	<0.00
**1. Population**								
1.1 Subgroup (diagnostic criteria)			0	0.41	R			
ICUD/ICS	2	242	85	0.00	R	2.68	(0.69,4.68)	0.00
Additional Diagnostic Criteria	4	224	77	0.00	R	1.67	(0.36,2.98)	0.01
1.2 Subgroup (severity)^#^			76.9	0.04	R			
Mild	3	269	58	0.09	R	1.46	(0.82,2.10)	0.00
Moderate	3	157	77	0.01	R	4.90	(1.72,8.08)	0.00
1.3 Subgroup(age)			0.44	0	R			
>39 years	3	284	74	0.02	R	1.79	(0.90.2.67)	<0.00
20–75 years	3	182	86	0.00	R	2.78	(0.39,5.18)	0.02
**2. Intervention**								
2.1 Subgroup (intervention types)			42.9	0.19	R			
Electroacupuncture	4	266	82	0.00	R	2.69	(1.11,4.27)	0.00
Manual acupuncture	2	200	86	0.00	R	1.11	(−0.61,2.83)	0.20
2.2 Subgroup (acupoints belong to)			0	0.83	R			
Bladder meridian	3	184	78	0.01	R	2.30	(0.58,4.03)	0.00
Bladder meridian combined with	2	122	88	0.00	R	2.58	(–0.28,5.43)	0.08
Non-Bladder Meridian (GV and CV)	1	160	/	/	R	1.91	(1.41,2.41)	0.00
2.3 Subgroup (duration)			0	0.50	R			
<45 days	3	182	86	<0.00	R	2.56	(0.37,4.75)	0.02
>45 days	3	284	72	0.03	R	1.73	(0.80,2.66)	0.00

*Data are presented *p*-Values for continuous variables comparison (Mean Difference). **Data are presented *p*-Values for Categorical variables comparison (Risk Ration). ***Data are presented *p*-values for ordered categorical variables comparison (Odds Ration). #In this study (33), patients with both mild and moderate urinary incontinence were included.

**Table 4:** Meta-analysis for PFMT combine with acupuncture.

Comparison of PFMT combine with acupuncture group and PFMT individually group								
Sample			Heterogeneity		Mode used	Meta-Analysis		
Outcomes	Studies	Patients	*I^2^*%	*p*		Mean Difference Odd/Risk Ration	95%CI	*p*
(B) ICIQ-SF*	5	406	75	0.00	R	2.63	(1.60,3.65)	<0.00
**1. Population**								
1.1 Subgroup (diagnostic criteria)			0	0.55	R			
ICUD/ICS	2	242	0	0.87	R	2.99	(2.37,3.61)	<0.00
Additional Diagnostic Criteria	3	164	87	0.00	R	2.26	(−0.03,4.55)	0.05
1.2 Subgroup (severity)^#^			33.6	0.22	F			
mild	3	269	0	0.70	F	2.83	(2.29,3.37)	0.00
moderate	2	97	2	0.31	F	3.77	(2.37,5.17)	<0.00
1.3 Subgroup(age)			0	0.49	R			
>39 years	3	284	31	0.23	R	2.93	(2.18,3.69)	<0.00
20–75 years	2	122	85	0.00	R	2.10	(−0.15,4.34)	0.07
**2. Intervention**								
2.1 Subgroup (intervention types)			8.6	0.30	R			
Electroacupuncture	3	206	4	0.37	R	3.04	(2.34,3.74)	<0.00
Manual acupuncture	2	200	93	0.00	R	1.47	(−1.39,4.33)	0.31
2.2 Subgroup (acupoints belong to)			0	0.71	R			
Bladder meridian	2	124	66	0.09	R	3.35	(1.37,5.33)	0.00
Bladder meridian combined with	2	122	85	0.00	R	2.10	(–0.15,4.34)	0.07
Non-Bladder Meridian (GV and CV)	1	160	/	/	/	2.87	(2.11,3.63)	0.00
2.3 Subgroup (duration)			0	0.94	R			
>45 days	2	224	0	0.62	R	2.75	(2.16,3.34)	<0.00
<45 days	3	182	84	0.00	R	2.66	(0.64,4.69)	0.01
(C) Self-reported***								
Therapeutic effect	4	321	0	0.89	F	3.79	(2.39,6.00)	<0.00

*Data are presented *p*-values for continuous variables comparison (Mean Difference). **Data are presented p-Values for Categorical variables comparison (Risk Ration). ***Data are presented *p*-values for ordered categorical variables comparison (Odds Ration).

## 4 Discussion

### 4.1 Main results

Based on high-quality evidence, the outcome of this study indicates that the application of EA could ameliorate SUI patients’ symptoms and enhance their quality of life, there were also no significant differences in adverse event reporting. Additionally, the application of EA combined with PFMT was more effective than using PFMT individually, and the within-group was still substantial in different subgroups. In summary, these conclusions support the use of electroacupuncture as a potential non-surgical treatment method to improve symptoms and quality of life in patients with SUI. However, to ensure the generalizability and applicability of these findings, future research is needed to further explore and validate the optimal treatment protocols and long-term effects.

### 4.2 What ideas does our research provide for clinical applications?

In contrast to the preceding meta-analyses (34, 35), the inclusion of the CNKI database search in our analysis was justified by the widespread use of acupuncture in China. Furthermore, it was essential to augment and enhance the evidence base, despite the fact that some of the studies included were of low quality. Our subgroup analysis indicated that the efficacy of acupuncture might be influenced by the severity of the disease, a finding that is corroborated by previous studies suggesting a correlation between the duration of urinary incontinence and the effectiveness of acupuncture (36).

Additionally, we observed that the effectiveness of manual acupuncture was slightly inferior to that of electroacupuncture, leading us to hypothesize that the efficacy might be related to electrical stimulation. In the subgroup analysis, no significant differences were found in either the subgroup of the bladder meridian combined with other meridians or the subgroup of manual acupuncture. This might be due to the manual acupuncture used in the study (30), which included mainly patients with mild to moderate stress urinary incontinence. We speculate that electroacupuncture could be more effective than manual acupuncture, especially for patients with moderate incontinence. However, this conclusion requires further research due to the limited number of studies available.

### 4.3 What progress does our research provide for future research?

Challenges still exist in the establishment of acupuncture control groups in the present clinical studies. Control acupuncture is mainly included without skin penetration and more shallow insertion of needles in suitable acupoints, acupuncture without skin penetration might not blind the participants in the placebo group adequately, and central *μ*-opioid receptor binding potential, which had a similar effect as acupuncture, could be increased if the non-acupoints were shallowly punctured (37, 38). The different of control acupuncture type indirectly affects the evaluation of acupuncture effect (37, 39). Compared with the previous meta-analysis (40, 41), this meta-analysis was placebo-controlled all were without skin penetration in the comparison of the electroacupuncture group, and the placebo control group may have reduced the potential for bias in the results. In addition to these, our study compared the differences before and after treatment, which may have reduced the potential bias caused by inconsistent baseline levels.

Nevertheless, all included studies were conducted in China, where satisfactory therapeutic effects might involve patients’ expectancies, beliefs, and the psychosocial context (42, 43). Multi-center validation of institutions in different countries and regions was requisite to guarantee the external validity of the study findings. Lastly, despite acupuncture had been considered safe in most studies (44, 45), it was an invasive procedure after all, and relevant adverse events would be reported as necessary, though over half of the included studies did not report it.

### 4.4 Strengths and limitations

Through detailed subgroup analyses based on inclusion criteria, intervention populations, and treatment duration, this study found that the efficacy of electroacupuncture combined with PFMT may be influenced by the severity of the condition. Due to the limited number of studies included, no significant intergroup differences were found between different types of acupuncture. However, it is speculated that the therapeutic effect of electroacupuncture might be superior to manual acupuncture. These results can assist physicians in selecting the most appropriate treatment methods for patients with varying degrees of SUI, especially when considering the use of electroacupuncture as an adjunctive treatment.

The study has several limitations, firstly, high heterogeneity was found in the comparison of acupuncture combined with PFMT, and this heterogeneity still persisted in subgroup analyses. The number of studies in this meta-analysis did not report details about the methodology. Based on the poor quality of the included studies, the sources of heterogeneity include not only clinically related heterogeneity, but methodology-related heterogeneity is also an essential factor of high statistical heterogeneity. The key point to high-quality evidence is to establish a study design with reliable evidence quality (46). Secondly, the publication bias cannot be assessed by funnel plot since the number of included studies was insufficient in the meta-analysis. Thirdly, the onset of SUI may be influenced by factors such as age, obstetric history, BMI, hormonal levels, chronic diseases, and lifestyle. We recommend that future research should provide detailed definitions and control over these factors. Doing so can enhance the quality and reliability of the studies, assist in identifying the SUI patient population most suitable for acupuncture treatment, and optimize treatment plans. This will contribute to improving therapeutic outcomes and providing stronger evidence to support clinical practices.

## 5 Conclusion

The results of this meta-analysis indicate that EA is more effective in improving the clinical symptoms and quality of life of patients with SUI compared to sham electroacupuncture, without increasing the risk of adverse events. Moreover, the therapeutic effect of SUI treated with electroacupuncture combined with PFMT yields a more positive response than PFMT alone. There are significant differences in treatment response among patients with varying severities of stress urinary incontinence, which deserves attention. Additionally, even though acupuncture is generally considered safe, detailed reporting on adverse events associated with acupuncture is necessary. Overall, electroacupuncture therapy alone or as an adjunctive treatment in combination with pelvic floor muscle training is beneficial for patients with stress urinary incontinence, offering additional clinical treatment options.

## Data Availability

The datasets used and/or analyzed during the current study are available from the corresponding author on reasonable request.
