# Supplementary material for: Efficacy and safety of acupuncture monotherapy or combined with pelvic floor muscle training for female stress urinary incontinence: a systematic review and meta-analysis
Source: Front Med (Lausanne). 2025 Jan 13;11:1499905. doi: 10.3389/fmed.2024.1499905 (PMC11771138; doi:10.3389/fmed.2024.1499905)
Supplement: Supplementary file 1 [file Data_Sheet_1.pdf]

Comparison between electroacupuncture and sham electroacupuncture

Urine leakage1 hour

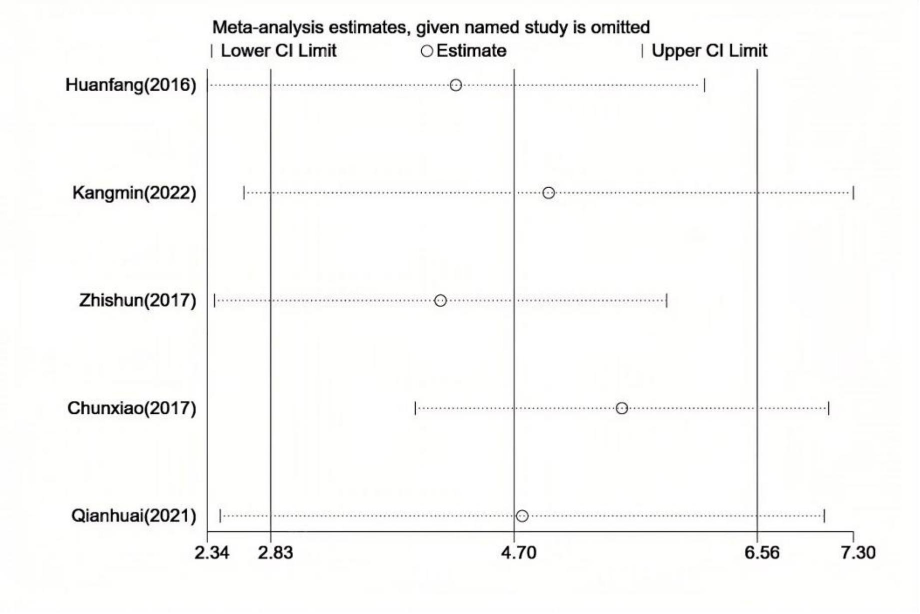

ICIQ-SF

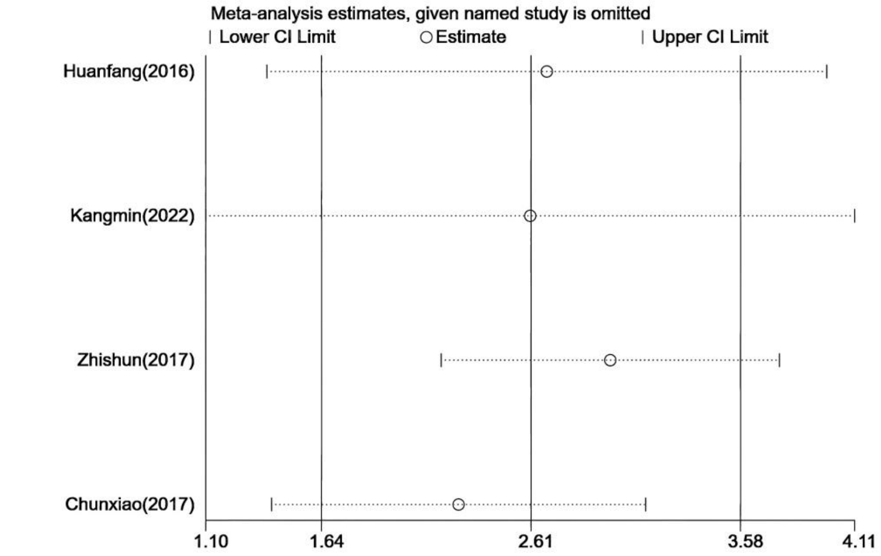

Comparison between acupuncture combine with PFMT and PFMT individually

Urine leakage1 hour

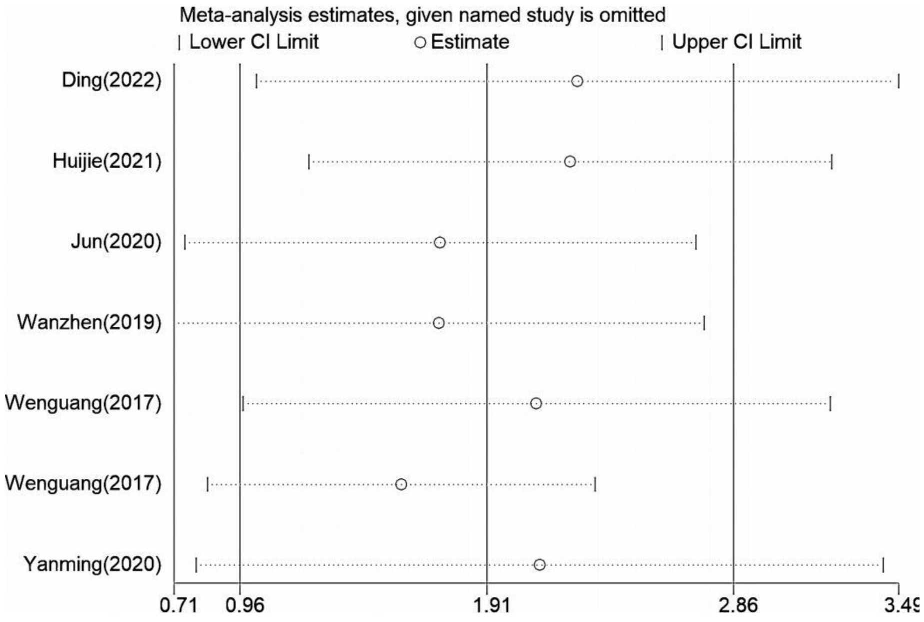

ICIQ-SF

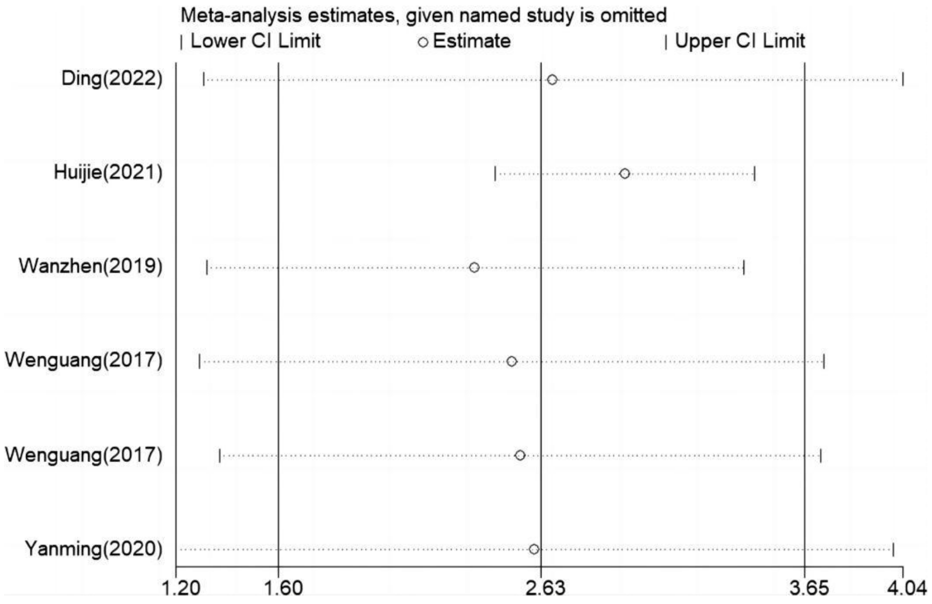

Supplement Figure1. Sensitivity analysis

Number	Pubmed	EMbase	Cochrane	CNKI
#1	("Urinary Incontinence, Stress/therapy"[Mesh]) OR (((Urinary Stress Incontinence[Title/Abstract]) OR (Incontinence, Urinary Stress[Title/Abstract])) OR (Stress Incontinence, Urinary[Title/Abstract]))	('stress urinary incontinence'/exp OR 'stress urinary incontinence' OR (('stress'/exp OR stress) AND urinary AND ('incontinence'/exp OR incontinence)))	Stress urinary incontinence'/Mesh	'Stress urinary incontinence'/subject
#2	("Acupuncture Therapy"[Mesh]) OR (((((((((Acupuncture Treatment[Title/Abstract]) OR (Acupuncture Treatments[Title/Abstract])) OR (Treatment, Acupuncture[Title/Abstract])) OR (Therapy, Acupuncture[Title/Abstract])) OR (Pharmacoacupuncture Treatment[Title/Abstract])) OR (Treatment, Pharmacoacupuncture[Title/Abstract])) OR (Pharmacoacupuncture Therapy[Title/Abstract])) OR (Therapy, Pharmacoacupuncture[Title/Abstract])) OR (Acupotomy[Title/Abstract])) OR (Acupotomies[Title/Abstract]))	'acupuncture'/exp/mj	Acupuncture'/Mesh	'Acupuncture'/subject
#3				
#4	(#1or#2) and #3（36）	#1 and #2（86）	#1 and #2（100）	#1 and #2（195）

Supplement Figure2. Search Strategy
